# Molecular Cloning, Overexpression and Characterization of a Novel Water Channel Protein from *Rhodobacter sphaeroides*

**DOI:** 10.1371/journal.pone.0086830

**Published:** 2014-01-31

**Authors:** Mustafa Erbakan, Yue-xiao Shen, Mariusz Grzelakowski, Peter J. Butler, Manish Kumar, Wayne R. Curtis

**Affiliations:** 1 Department of Biomedical Engineering, Pennsylvania State University, University Park, Pennsylvania, United States of America; 2 Department of Chemical Engineering, Pennsylvania State University, University Park, Pennsylvania, United States of America; 3 AquaZ Inc, Cincinnati, Ohio, United States of America; Technion-Israel Institute of Technology, Israel

## Abstract

Aquaporins are highly selective water channel proteins integrated into plasma membranes of single cell organisms; plant roots and stromae; eye lenses, renal and red blood cells in vertebrates. To date, only a few microbial aquaporins have been characterized and their physiological importance is not well understood. Here we report on the cloning, expression and characterization of a novel aquaporin, RsAqpZ, from a purple photosynthetic bacterium, *Rhodobacter sphaeroide*s *ATCC 17023.* The protein was expressed homologously at a high yield (∼20 mg/L culture) under anaerobic photoheterotrophic growth conditions. Stopped-flow light scattering experiments demonstrated its high water permeability (0.17±0.05 cm/s) and low energy of activation for water transport (2.93±0.60 kcal/mol) in reconstituted proteoliposomes at a protein to lipid ratio (w/w) of 0.04. We developed a fluorescence correlation spectroscopy based technique and utilized a fluorescent protein fusion of RsAqpZ, to estimate the single channel water permeability of RsAqpZ as 1.24 (±0.41) x 10^−12^ cm^3^/s or 4.17 (±1.38)×10^10^ H_2_O molecules/s, which is among the highest single channel permeability reported for aquaporins. Towards application to water purification technologies, we also demonstrated functional incorporation of RsAqpZ in amphiphilic block copolymer membranes.

## Introduction

High water transport rates across some biological membranes such as renal collecting ducts and red blood cell plasma membranes were not well understood until the discovery of the first water channel protein in red blood cells [1]. Many aquaporins have subsequently been discovered in many genera including invertebrates [2], plants [3] and microorganisms [4]. Aquaporins retain ions (including protons) and molecules and are either solely permeable to water (orthodox aquaporins) or small uncharged molecules such as glycerol (glycerol facilitators) [5]. The high permeability and water selectivity of aquaporins is due to two main structural features common to most aquaporins. The first feature is an hourglass structure with six membrane-spanning domains joined by three extracellular and two intracellular loops. Two of these loops (one being extracellular, one being intracellular) carry the Asparagine-Proline-Alanine (NPA) motif that is preserved in most aquaporins. These loops dip back into the membrane to form a continuous water channel (∼2.8 Å in diameter), through which water molecules diffuse along the osmotic gradient [6]. A second feature is positively charged arginine residues, which repel positively charged solutes, in close proximity to the NPA motifs, where additional histidine residues contribute to size restriction. The narrowest region in the channel is approximately 2.8 Å in diameter [5]. Water undergoes conformational changes within the channel by making hydrogen bonds with the same arginine residues, which prevents proton transport by breaking the proton wire [7]. A final notable feature is the homotetrameric structure of orthodox aquaporins that was initially revealed by electron microscopy studies on red blood cells [8] and proteoliposomes, and subsequently confirmed by high-resolution structures obtained by X-ray and electron crystallography [9].

Higher organisms including vertebrate, invertebrate, and plants have multiple copies of aquaporin genes to regulate water transport activities in different tissues. The regulation and function of these gene products are well understood in many cases, e.g. controlled regulation of AQP2 in renal collecting duct [10]. In the case of microbial aquaporins, however, there is a lack of understanding of their physiological role(s), since diffusive water permeability through the plasma membrane of single cell organisms is sufficiently large to mediate cell growth and response to environmental changes in medium osmolarity [11]. Indeed, only 10 percent of the prokaryotic organisms, whose whole genome sequence is available, have aquaporin homologs [11]. Moreover, aquaporin knockout mutants were frequently not associated with a noticeable change in phenotype suggesting that aquaporins were not essential for these organisms under given growth conditions [12]. However, reports have provided contradicting results or revealed specialized roles. In one study on *E. coli* aquaporin, EcAqpZ, a reduction in growth rate was reported for knockout mutants indicating EcAqpZ mediated water uptake in the logarithmic growth phase [13]. Archeal aquaporin, AqpM, from *M. marburgensis* was shown to be functionally expressed and responsible for cytoplasmic shrinkage of the cell exposed to hypertonic shock [14]. A recent study showed that the aquaporin of cyanobacterium, *Synecoccous sp. PCC7942*, transports CO_2_ as well as water and has an apparent role in CO_2_ uptake during photosynthesis [15]. Aquaporins from S. *cerevisiae,* AQY1 and AQY2 were shown to improve the freeze tolerance of yeast under rapid freezing conditions [16].

*Rhodobacter sphaeroides* is a purple non-sulphur photosynthetic bacterium, commonly studied as a model organism in photosynthesis research. It is characterized by a wide capability for growth modes including aerobic and anaerobic respiration, fermentation, photoheterotrophy and chemolithotrophy. In the photosynthetic growth mode, membrane surface area of *R. sphaeroides* increases extensively due to biosynthesis of intracellular membranes accommodating the photosynthetic apparatus. These features have been used to employ *R. sphaeroides* in expression of membrane proteins for structural and functional studies [17], [18], [19]. In this study, we have cloned, overexpressed and purified a novel aquaporin from *Rhodobacter sphaeroides ATCC 17023* (RsAqpZ) utilizing a native expression system. The protein was fully functional, as determined by stopped-flow light scattering experiments and showed high water permeability as well as low activation energy, similar to the well-studied *E. coli* homolog (EcAqpZ). However, higher insertion efficiency was observed for this protein at higher protein to lipid weight ratios (PLR) starting from 0.005. The large decrease in permeability of proteoliposomes with high PLR in some aquaporin studies, for example Borgnia et al. [20], can be explained using the direct insertion efficiency measurement technique developed in this study, which involves utilization of a fluorescent fusion protein to determine the protein stoichiometry per liposome via fluorescence correlation spectroscopy.

Aquaporins (in particular EcAqpZ) are being studied extensively for application to biomimetic desalination membranes and their scale-up is quite challenging [21]. In these studies EcAqpZ is commonly inserted in block copolymer vesicles due to the improved stability of polymers as compared to lipids [22]. We show that RsAqpZ also remains active in block copolymer membranes and can be packed at high densities. Using the photoheterotrophic *Rhodobacter* expression platform, we demonstrate a high level expression (∼20 mg protein/L culture) of functionally active and highly permeable RsAqpZ and show that it remains active in lipids and amphiphilic block copolymers.

## Materials and Methods

### Materials

n-octyl β-D-glucoside (OG) was purchased from Affymetrix, Inc., Santa Clara, CA. Phosphatidylcholine (chicken egg, PC) and phosphatidylserine (porcine brain, PS) were purchased from Avanti Polar Lipids, Inc., Alabaster, AL. Polyclonal His-tag antibody and anti-rabbit-HRP antibody were purchased from GenScript USA, Inc., Piscataway, NJ. Co-NTA resin was purchased from Thermo Fisher Scientific, Rockford, IL. All other chemicals were purchased from Sigma Aldrich or Thermo Fisher Scientific. Restriction enzymes, DNA polymerases, ligase and markers were purchased from New England Biolabs.

### Amino Acid Sequence and Phylogeny Analysis

Aquaporin amino acid sequences were retrieved from Genbank for *Rhodobacter sphaeroides* ATCC 17023 (ABA78939.1), *Escherichia coli K12* (EcAqpZ, BAA08441.1 and GlpF, AFH13815.1), *Methanobacter marburgensis* (AqpM, ADL58146.1), *Synecoccocus elongatus* (SsAqpZ, AAM82672.1), *Pichia pastoris* (AQY1, CCA39392.1), *Spinacia oleracea* (SOPIP2;1, AAA99274.2) and *Homo sapiens* (AQP1, NP_932766.1; AQP4, AAH22286.1; AQP9, NP_066190.2). The alignment was performed using Clustal-Ω multiple sequence alignment software [23]. A phylogeny tree file based on the Neighbor-joining method [24] was created using Clustal W2 [23]. The rooted phylogeny tree was constructed using MEGA5 [25].

### Strains, Plasmids and Growth Conditions

*R. sphaeroides* has been used as a membrane protein expression platform due to its abundant inducible intracellular membranes (ICM) accommodating the photosynthetic apparatus, which is comprised of core complexes (photosynthetic reaction center [RC] and first tier light harvesting antenna [LHI]) and peripheral complexes (second tier light harvesting antenna [LHII]) [17]. Since LHII can account for as much as 10% of the cell biomass under anaerobic photoheterotrophic growth conditions [26], we predominantly used a LHII knockout strain, *PUC705-BA* [27], to increase the membrane area available for recombinantly expressed RsAqpZ. Expression vector, *pRKPLHT7*, was a derivative of broad-host-range plasmid *pRK404* [*26]*, *[28*], in which expression of recombinant RsAqpZ is under control of the native *puc* promoter. In *Rhodobacter*, *puc* promoter controls the expression of the *puc* operon, which encodes for LHII complex and gets activated by low light intensity as well as reduced oxygen tension [27].

Wild type strain, *R. sphaeroides* ATCC 17023 [29], was used as a template to PCR amplify the *RsAqpZ* gene. *RsAqpZ* was expressed from a *pRKPLHT7*-based plasmid in the *PUC705-BA* strain. *PUC705-BA* bearing the empty *pRKPLHT7* plasmid was used as a negative control in expression screening with western-blot. Unless otherwise noted, *R. sphaeroides* strains were cultivated in minimal (MR26) [30] or complex medium (YCC) [31] under semi-aerobic or photoheterotrophic conditions with 1 µg/mL tetracycline as required for selection [17]. Variations in antibiotic use are described in a subsequent section.

### Cloning of Rhodobacter AqpZ

A BLAST search performed on the genome sequence of *Rhodobacter sphaeroides 2.4.1* revealed a single putative aquaporin gene (RSP_2782) showing 44% identity and 58% similarity to EcAqpZ. We designed forward (^5′^CCACGGACTAGTGGAGGCCATTCATGACCAAGAAGCTC^3′^) and reverse primers (^5′^TAAATAAGATCTGAGCGTGGCCGCGCCGGTTGCGGG^3′^) to amplify this gene sequence from genomic DNA of *R. sphaeroides ATCC 17023* and cloned it into the *pRKLHT7* expression vector between SpeI and BglII restriction sites. The cloning strategy is described in File S1 and Figure S1. Immediately downstream of SpeI restriction site and six base-pairs upstream of the start codon of *RsAqpZ*, a ribosome binding site (GGAGG) exists that is highly conserved in the photosynthetic gene cluster of *R. sphaeroides*. This RBS was included to provide a bias towards protein overexpression that is observed for the native light-harvesting genes [32]. The vector was transformed into *E.coli DH5α* by electroporation. After selection of transformants in the presence of 15 µg/mL tetracycline (tet), the transfer of *pRKLHT7-RsAqpZ* plasmid was verified by colony PCR. The plasmid sequence was confirmed by DNA sequencing.

Plasmid DNA transfer into *Rhodobacter* has not been achieved for the *pRK404* - based plasmids by chemical or electroporative transformation, likely due to prolific restriction endonuclease activity [33]. For this reason, conjugative transformation employing a conjugation competent strain of *E.coli, S17-λ-pir* [34], was used to mobilize *pRKLHT7-RsAqpZ* plasmid into *PUC570BA. Rhodobacter* exconjugates were selected on a proline dropout media, MR26 [26], over *S17-λ-pir,* and grown photo-heterotrophically [34].

### Protein Expression and Purification

*R. sphaeroides* was grown under photoheterotrophic conditions using high power IR-LEDs (Snow Dragon Industrial Co., Ltd, Shenzhen, China) with a peak emission wavelength of 850 nm at a light intensity of approximately 4 W/m^2^. It was recently shown that low light intensity increases ICM biosynthesis and expression of LH2, which is controlled by *puc* promoter driving the expression of recombinant proteins in our expression system [35]. A single colony of *Rhodobacter* picked from a YCC agar plate containing 1.5 µg/mL tet was inoculated into a 50 mL conical tube containing 15 mL YCC+tet medium. After 24 hrs of growth at 34°C with shaking at 200 rpm, the inoculum was transferred in a 250 mL Erlenmeyer flask containing an additional 50 mL of fresh YCC media with 1 µg/mL tetracycline to culture for another 24 hr at 34°C, 100 rpm. Anaerobic photoheterotrophic growth was fully implemented by transferring 50 mL of the semi-aerobic culture into the T-flask (# 431080, Corning) and topped off with approximately 700 mL YCC media with 1 µg/mL tet. The culture in T-flask was placed horizontally below an IR-LED array, while the culture temperature was maintained at 25°C by partial submergence in a simple plastic water bath on top of a stir plate to provide agitation from the stir bar inside the T-flask. A miniature axial box fan blowing across the T-flask also helped to maintain temperature by dissipating the radiant heat of the IR panel.

*E.coli JM109* cells harboring an N-terminal 10-His-tagged version of *E.coli K12* Aquaporin Z were grown following a previously published procedure [20]. Briefly, a 10 mL inoculum was prepared from a single colony growing the cells overnight in LB media with 100 µg/mL ampicillin. This was then used to inoculate a 1 L culture in the same medium in a 4 L Erlenmeyer flask. This culture was grown overnight and then induced with 1 mM IPTG for 8 hrs.

*R. sphaeroides* and *E. coli* cells were harvested by centrifugation at 8000 rpm for 30 min and frozen at least one hour at −80°C prior to cell lysis. Cell lysis was performed in a buffer containing 100 mM K_2_HPO_4_, 1 mM MgSO_4_, 10 mM imidazole, 1 mM PMSF, 0.1 mg/ml DNase I, 0.02 mg/ml RNase, Halt protease inhibitor cocktail (Pierce), 1 mg/ml lysozyme at pH = 7.0 by sonication 3 times at 4°C in pulsed mode for 3 min at 50% duty cycle using a horn sonicator (Sonifier Cell Disruptor 350, Branson Ultrasonics Corp., Danbury, CT). Cells were then incubated at 37°C for 1 hr and sonicated again for 3 times. Cell debris and unbroken cells were pelleted at 18,000×g, 4°C for 30 min and the supernatant was centrifuged at 240,000×g for 2 hrs to pellet the membrane fraction. The supernatant was removed and membrane fraction was solubilized overnight in a buffer containing 5% n-octyl-β-D-glucopyranoside (OG), 100 mM K_2_HPO_4_ (pH = 7.0), 200 mM NaCl, 10% glycerol, 20 mM imidazole, and 2 mM β-mercaptoethanol (BME). Insoluble material was pelleted at 240,000×g, 4°C for one hour and supernatant was incubated with His-pur Co-NTA resin (Pierce) for 2 hrs with gentle agitation. The resin was loaded onto an affinity column, and the column was washed with up to 30 bed volumes of buffer containing 100 mM K_2_HPO_4_ (pH = 7.0), 1% OG, 200 mM NaCl, 10% glycerol, 50 mM imidazole, and 2 mM BME. The protein was eluted with 100 mM K_2_HPO_4_ (pH = 7.0), 1% OG, 200 mM NaCl, 10% glycerol, 500 mM imidazole, and 2 mM BME. Sample purity and loss was estimated by running eluted fractions on a SDS-PAGE gel.

### Polymer Synthesis

The triblock copolymer (ABA55) consisting of hydrophilic poly-2-methyloxazoline (PMXOA_8_) coronal blocks and hydrophobic poly-dimethylsiloxane (PDMS_55_) core block was synthesized as described previously [36]. The hydrophobic block was synthesized via acid catalyzed polycondensation of dimethoxydimethylsiloxane in presence of 1,3- bis (4-hydroxybutyl) tetramethyldisiloxane (45∶1). Triflate-activated polydimethylsiloxane macro-initiator was used to initiate ring-opening polymerization of 2-methyl-oxazoline according to the procedure described elsewhere [36]. The number average molecular weight of block copolymers averaged at 5430 g/mol as determined by ^1^H NMR. Specific block composition was found to be PMOXA_8_PDMS_55_PMOXA_8_. GPC analysis in chloroform, using polystyrene calibration, revealed a polydispersity index (PDI) value of 1.34 (Instrument: Waters 717 Plus Auto Sampler, 2414 RI Detector, 1515 Isocratic HPLC Pump, in-line degasser AF, 150 uL injection volume 40 Minute Run time, 1 mL/min solvent flow rate).

### Reconstitution of RsAqpZ and EcAqpZ in Liposomes and Polymersomes

The functionality of the purified RsAqpZ and EcAqpZ was evaluated by measuring osmotic water permeability of proteoliposomes embedded with purified protein as described elsewhere [20]. Briefly, following metal affinity purification, protein concentration was measured by the Bradford method. RsAqpZ was then mixed with natural phospholipids, phosphatidylcholine (egg PC) and phosphatidylserine (porcine PS) (molar ratio of 4∶1), at varying protein to lipid ratios (PLR) in a buffer containing 20 mM HEPES, 100 mM NaCl and 0.02% NaN_3_. This mixture was placed in 350 µL dialysis buttons (Hampton Research, Aliso Viejo, CA) covered with a 12–14 kDa MWCO dialysis membrane (Spectra/Por 2, Spectrum Laboratories Inc., Rancho Dominguez, CA) and dialyzed for four days at 4°C to remove detergent and form proteoliposomes. For pH experiments, buttons were moved into different buffers with pH values between 4.5 and 8.5 and dialyzed for two extra days to equilibrate pH within the vesicles. Buffering agents used at different pH values were 20 mM sodium acetate for pH 5.0; 20 mM MES for pH 6.0; and 20 mM HEPES for pH 7.0, 7.4 and 8.0. Proteoliposomes were extruded through a 200 nm track-etched nucleopore membrane (Nucleopore, Whatman Ltd., Maidstone, Kent, UK) to obtain unilamellar vesicles with low polydispersity index (PDI <0.2).

ABA55-RsAqpZ and ABA55-EcAqpZ proteopolymersomes were formed by film rehydration [37]. Briefly, 8 mg of block copolymer was dissolved in 2 mL chloroform in a round-bottomed flask and the solvent was evaporated using a rotary evaporator. The resulting thin film was further dried under high vacuum overnight in a vacuum desiccator (Labconco, Kansas City, MO). In order to obtain proteopolymersomes at different molar protein to polymer ratios (PrPR), purified RsAqpZ and EcAqpZ were diluted in 2 ml sample buffer containing 20 mM HEPES (pH 7.4), 1% OG, 100 mM NaCl and 0.02% NaN_3_ and added on the film followed by a 3 hr incubation for rehydration of the film. The flask was placed on a stir plate and the protein/polymer/detergent ternary mixture was stirred overnight. The mixture was transferred into a Slide-A-Lyser dialysis cassette (10 kDa MWCO, Thermo Scientific) and dialyzed against the sample buffer without OG for 2 days until proteopolymersomes were formed. The sample was extruded 10 times through a 0.4 µm and 10 times through 200 nm track-etched filters (Whatman Ltd., Maidstone, Kent, UK) to obtain unilamellar, monodisperse vesicles. The size and PDI of proteoliposome and proteopolymersome samples were measured in a dynamic light scattering instrument (Zetasizer Nano, Malvern Instruments, Worcestershire, UK).

### Osmotic Water Permeability Experiments

Aquaporin-mediated water transport across proteoliposomes was measured in a stopped-flow light scattering device (KinTek SF-300X, Snowshoe, PA) and compared to control liposomes prepared identically but devoid of RsAqpZ. Liposomes were rapidly mixed with its corresponding dialysis buffer consisting of additional 0.2M NaCl to provide an outwardly directed gradient to drive water transport outside of the liposome. This exposure to hypertonic solution results in shrinkage of the vesicles and increase in light scattering at 90° according to the Rayleigh-Gans theory [38], which was adapted and used for liposome and polymersome samples [39]. Stopped flow data was fit to a double exponential rise equation [20] using Matlab Curve Fitting Toolbox to obtain the osmotic water permeability rate constant, *k*. Equation 1 is used to calculate water permeability for empty and RsAqpZ-loaded vesicles:
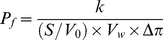
(1)where, *P_f_* is water permeability; *k* is rate constant; S/V_0_ is initial surface to volume ratio of liposome; V_w_ is molar volume of water, 18 cm^3^; Δπ is osmotic pressure gradient.

### Measurement of Single Channel Permeability

We created a C-terminal fusion of RsAqpZ protein with mBanana fluorescent protein [40] by modifying the original *pRKPLHT7* expression vector. This construct was codon optimized for *Rhodobacter* expression by GenScript (Piscataway, NJ). The purified fusion protein was used in a fluorescence correlation spectroscopy (FCS) based method to determine the average number of RsAqpZ-mBanana molecules incorporated per proteoliposome. To obtain proteoliposomes that are free of non-incorporated RsAqpZ, a final separation from non-incorporated protein was achieved by gel filtration chromatography using a Superdex 200 column with a buffer containing 20 mM HEPES, 100 mM NaCl and 0.02% NaN_3_. Column fractions rich in proteoliposomes were collected and pooled for stopped-flow light scattering and FCS experiments.

FCS experiments were performed on a time correlated single photon counting (TCSPC) module (Becker-Hickl GmbH, Berlin, Germany). The detailed instrumentation setup is described elsewhere [41]. The light source was a Nd:YAG pulsed laser with an emission maximum of 532 nm. A 60x water immersion objective with a numerical aperture of 1.2 was used to focus the laser beam into a diffraction-limited focal point 40 µm above the cover slip within the sample. Laser power was set to 5 µW/m^2^ by measuring light intensity at the back of the objective aperture. The dimensions of the confocal volume were determined using a fluorescent dye standard with known diffusion coefficient (Rhodamine B). Proteoliposome samples were diluted in HEPES buffer containing a final concentration of 2.5% OG. Data collection was kept under 3 minutes to minimize background interference. The number of RsAqpZ-mBanana tetramers embedded per proteoliposome was calculated by taking the ratio of number of particles in confocal volume before and after solubilization of proteoliposomes, which is a method that has recently been reported elsewhere for a potassium channel [42] and a bacterial translocon [43]. The detailed procedure employed for our study is described in further detail in the results section as it was based on several insights obtained during the testing.

After measurement of the water permeability for the proteoliposomes and control liposomes by stopped-flow experiment, total water permeability contributed by RsAqpZ in proteoliposomes was calculated. Knowing the average number of tetramers per liposome, the subunit permeability of RsAqpZ was determined by combining the results from stopped-flow light scattering and FCS experiments.

## Results

### Amino Acid Sequence and Phylogeny Analysis

**Figure 1:**
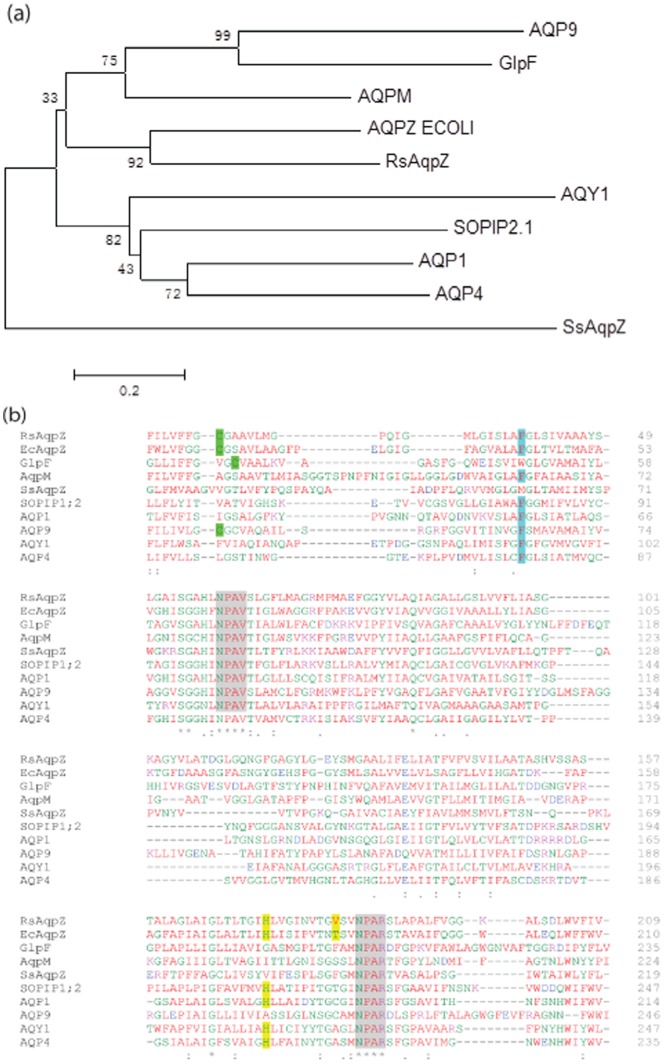
Evolutionary relationship and amino acid sequence alignment of RsAqpZ with other aquaporin channels. a) Phylogeny analysis of RsAqpZ indicates that it is an orthodox aquaporin with a close relationship to *E.coli* AqpZ, as well as the archeal aquaporin, AqpM. Evolutionary relationship of RsAqpZ to other aquaporins was evaluated by the Neighbor-Joining method [24]. The percentage of replicate trees in which the associated taxa clustered together in the bootstrap test (2000 replicates) is shown next to the branches [44]. The evolutionary distances were computed using the Equal Input method [45] and are in the units of the number of amino acid substitutions per site. The analysis involved 10 amino acid sequences. All ambiguous positions were removed for each sequence pair. Evolutionary analyses were conducted in MEGA5 [25]. b) Multiple sequence alignment of aquaporin sequences. Amino acid sequence alignment was performed using Clustal Ω [23] with the mRNA translation sequences retrieved from Genbank. RsAqpZ had signature residues Phe-39, His-173 and Arg-188 representing the orthodox aquaporins. Val-182 is a unique residue in the selectivity filter of RsAqpZ, which has significantly higher hydrophobicity, compared to the equivalent Thr-183 of EcAqpZ and Cys-189 of AQP1. Similar small hydrophobic residues were also found in AQY1 from *Pichia pastoris* (Ala-220) and human AQP4 (Ala-209), which might contribute to relatively higher water permeability in these aquaporins due to reduced interaction between water molecules and the amino acid residue in the selectivity filter.

Phylogeny analysis suggests that RsAqpZ is an orthodox aquaporin with a close relationship to *E.coli* AqpZ, as well as the archeal aquaporin, AqpM. It is located in a cluster that is distinct from the aquaglyceroporins, *E.coli* GlpF and human AQP9 (Figure 1A). The newly reported cyanobacterial aquaporin from *Synechococcus,* SsAqpZ [15] fell into a distinct category from the rest of the aquaporins.

Multiple sequence alignment of RsAqpZ with other aquaporins showed that most aquaporin ‘signature sequences’ are conserved between RsAqpZ and EcAqpZ. Cys-20 is the residue responsible for tetramerization of EcAqpZ, and is conserved in RsAqpZ. Phe-43, His-174, Thr-183 and Arg-189 amino acid residues form the channel selectivity filter of EcAqpZ. Except for Thr-183, the remaining three key residues are conserved in RsAqpZ. In RsAqpZ, threonine is replaced by valine (Val-182), which provides a significantly higher hydrophobicity index. Earlier studies with Thr183 Cys mutant of EcAqpZ did not show any difference in water transport [46]. However, the highly hydrophobic valine residue present in RsAqpZ, in contrast to the hydrophilic threonine residue in EcAqpZ, might reduce interaction between water molecules and the protein which could explain the difference in water transport rate described later in this study.

### Cloning, Expression and Purification of *Rhodobacter AqpZ*

**Figure 2:**
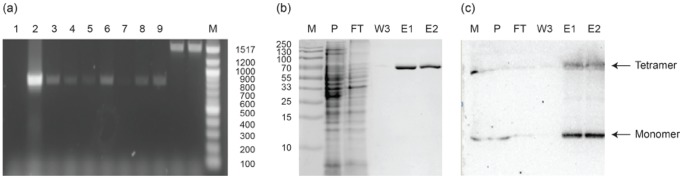
Cloning and verification of RsAqpZ expression. a) Verification of the transfer of RsAqpZ into *Rhodobacter sphaeroides 17023* by colony PCR using primers directed to *pRKPLHT7* vector backbone. Lanes 1: negative control, 2: positive control (*E. coli* cloning vector), 3–9: *Rhodobacter* exconjugants (expected band 864 bp), L: NEB 100 bp ladder; b) SDS-PAGE of RsAqpZ overexpressed and purified from *Rhodobacter*. M:Marker, P:Pellet, FT: Flow-through, W3∶3rd Wash fraction, E1: First Elution from IMAC column, E2: Second elution; c) Western-blot image of the gel in part b); RsAqpZ in monomeric and tetrameric form were detected by anti-His antibody.

The *RsAqpZ* gene (Genbank ID: CP000143) was amplified from genomic DNA of *Rhodobacter sphaeroides* ATCC 17023 by colony PCR. This amplicon of 764 bp was then cloned into the multiple cloning site of *pRKPLHT7* expression vector using SpeI and BglII sites. The plasmid, *pRKPLHT7-RsAqpZ*, was maintained in *E. coli* DH5α strain and transformed into *E. coli S17-λ-pir* for mobilization into *Rhodobacter* by conjugation. *Rhodobacter* exconjugants were verified by colony PCR (Figure 2a).

RsAqpZ was purified from the visible purple membrane fraction of photoheterotrophically grown *Rhodobacter* cells using cobalt metal (Co) affinity chromatography at 5 mg recovered protein/L; higher expression levels of up to 20 mg/L could be purified at a higher yield using Ni-NTA resin but consistently higher purity was obtained with Co-NTA resins and therefore utilized for the remainder of this study. 5 and 0.5 µg of protein eluted from the Co column was run on a 12% polyacryamide gel for SDS-PAGE and western-blot experiment, respectively. The majority of RsAqpZ remained in tetrameric form as detected around 70 kDa in SDS-PAGE gel, where the monomer band was not noticeable (Figure 2.b). However, western-blot analysis revealed both monomeric and tetrameric bands around 17 kDa and 70 kDa, respectively (Figure 2.c). It should be noted that a deviation of the observed gel molecular weight from expected physical molecular weights is a common observation for membrane proteins due to unusual SDS binding stoichiometry [20], [47].

### Osmotic Water Permeability Experiments

**Figure 3:**
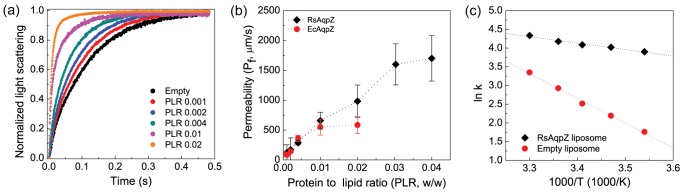
Biophysical characterization of RsAqpZ. a) Stopped-flow light scattering of PC/PS vesicles (4∶1 molar ratio) with and without RsAqpZ. RsAqpZ proteoliposomes were formed at varying w/w protein to lipid ratios (PLRs). Osmotic water permeability rate constant, k, is obtained by fitting the initial rise of the curve to a double exponential model, b) Water permeability of vesicles embedded with RsAqpZ and EcAqpZ at different PLR. Osmotic water permeability, *P_f_*, is calculated using Equation 1. The average liposome diameter was measured by dynamic light scattering. c) Arrhenius plot; rate constants of RsAqpZ proteoliposome shrinking and control vesicles was measured at different temperatures and Arrhenius activation energies were calculated by the slope of the trendline.

Purified RsAqpZ was incorporated into PC/PS vesicle membranes using the detergent dialysis method. The average diameter of the vesicles was 149.6±15.8 nm (n = 48) as determined by dynamic light scattering. Water permeability of proteoliposomes embedded with RsAqpZ and EcAqpZ as well as empty liposomes was measured in a stopped-flow light scattering apparatus. An outwardly directed 0.2M NaCl gradient was imposed on the proteoliposomes and change in light scattering due to vesicles shrinkage was monitored on a millisecond time scale (Figure 3.a). The results from these experiments clearly demonstrated the functionality of RsAqpZ showing more than order of magnitude higher osmotic water permeability for RsAqpZ embedded proteoliposomes as compared to control liposomes devoid of RsAqpZ.

The rate of change in light scattering was proportional to the protein concentration in the vesicles from a w/w protein to lipid ratio (PLR) from 0.001 up to a PLR of 0.01 and 0.02 for EcAqpZ and RsAqpZ, respectively (Figure 3.b). RsAqpZ achieved the highest water permeability at 1700.1±377.7 µm/sec at a PLR of 0.04. By comparison, the highest permeability observed for the *E. coli* aquaporin was 586.2±141.7 µm/sec that occurred at a lower protein loading (PLR = 0.02). We observed visible precipitation for EcAqpZ samples during dialysis, which implied lower incorporation efficiency leading to lower water permeability compared to RsAqpZ samples at the same PLR (Figures S2 and S3). The average water permeability of empty PC/PS vesicles was 95.4±30.5 µm/sec. The Arrhenius activation energy associated with water transport can be assessed based on temperature-dependent permeability rate constants (k). Osmotic shrinking experiments were repeated for empty liposomes and proteoliposomes with PLR 0.01 at different temperatures to produce an Arrhenius plot (k versus 1/T). The energy of activation for water transport is calculated as 2.93±0.60 kcal/mol and 12.39±2.21 kcal/mol, for aquaporin-loaded and empty liposomes respectively (Figure 3.c).

We evaluated a possible pH gating property of RsAqpZ by measuring water permeability at different pH values (Figure S4.). No statistically significant change in water permeability was observed between pH values of 5 and 8.

### Measurement of Single Channel Water Permeability

**Figure 4:**
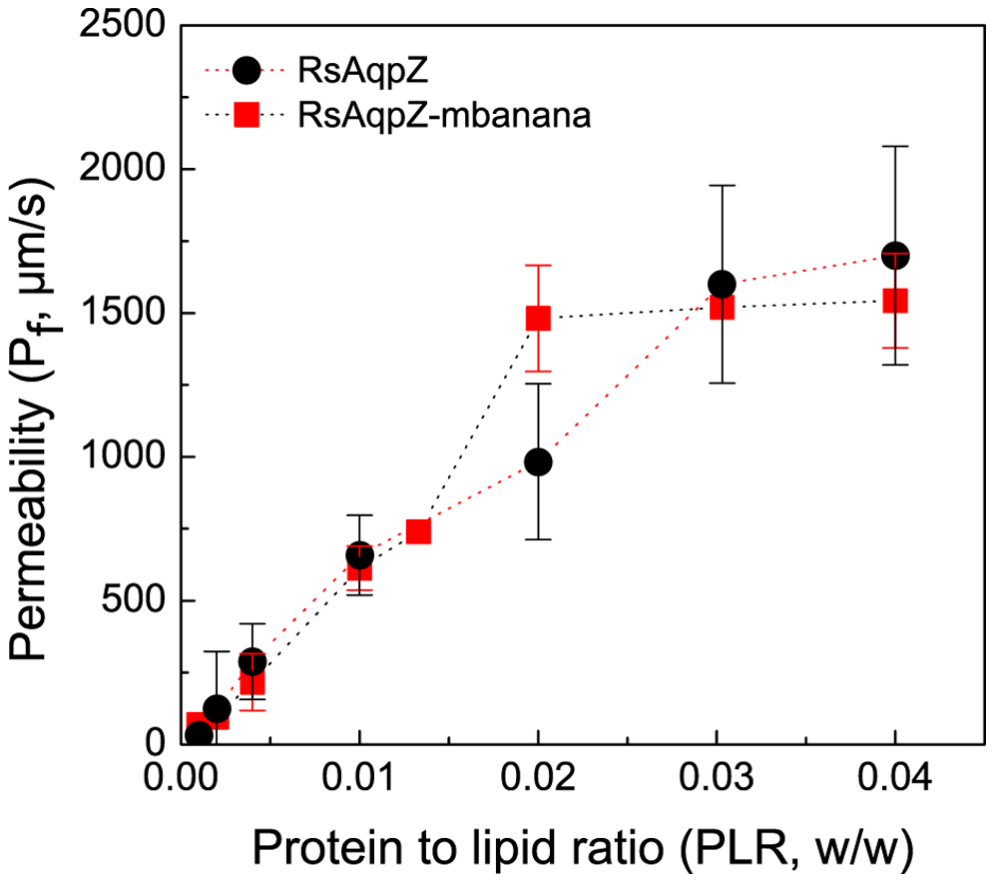
Comparison of water channel activity for RsAqpZ and RsAqpZ-mBanana in phospholipid vesicles. mBanana fluorescent protein was expressed as a C-terminal fusion partner along with RsAqpZ. The water permeability did not show a significant change, which enabled assessment of the protein properties at the single molecule level.

Although aquaporin single channel transport rates have been reported in the literature, these have assumed 100% incorporation of the purified protein into liposomes [20] or estimated insertion efficiency using techniques such as western blotting [48], which may induce substantial error due to inconsistencies with protein loading in polyacrylamide gel, transfer of the proteins from polyacrylamide gel onto nitrocellulose (or PVDF) membrane and other experimental variables [49]. As can be deduced from Figure 3.c, the water permeability initially increases in proportion to the protein amount provided in the proteoliposomes. However the permeability of EcAqpZ saturated at a certain PLR suggesting the efficiency of protein incorporation into lipid bilayers was significantly reduced. This observation clearly demonstrates the need for an accurate method for determination of the single channel water permeability of aquaporins. In order to facilitate more precise measurements of protein levels, we re-cloned RsAqpZ with a C-terminal mBanana fluorescent protein tag (RsAqpZ-mBanana). mBanana is a (27 kDa) fluorescent protein (DsRed variant) with absorption and emission maxima of 540 and 550 nm, respectively [40]. Since the permeability values are based on stopped flow measurements of a given proteoliposome preparation, it is important that the presence of the fluorescent tag should not alter water permeability. Indistinguishable permeability for the C-terminally tagged RsAqpZ-mBanana is shown in Figure 4, where the mass PLR ratio reflects the appropriate correction for the increase in protein mass due to the tag. This convention of considering only the RsAqpZ portion of the protein is adopted throughout the paper so that there is a consistent PLR basis for comparing RsAqpZ-mBanana and RsAqpZ proteoliposomes.

In an FCS experiment, time-dependent changes in fluorescent intensity within a small observation (confocal) volume (∼ 1 femtoliter) are monitored and the fluctuations are fit to an autocorrelation function described below (Equation 2).
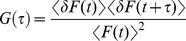
(2)


Here, G(τ) is the normalized autocorrelation function; δF(t) is the fluorescence intensity fluctuation at time t; δF(t+τ) is the fluorescence intensity fluctuation after a time lag τ, and F(t) is the average fluorescence intensity at time *t*. When τ = 0, the term on the right side of the Equation 2 equals the variance of the fluorescence intensity fluctuation, which yields G(0) = 1/N. N represents the average number of fluorophores in the confocal volume. These principles were used to obtain the concentration of mBanana-tagged RsAqpZ tetramers in aqueous solutions and in proteoliposomes by fitting observed autocorrelation curves from FCS experiments to a 3D diffusion model shown below in Equation 3 [41].
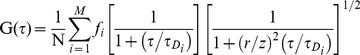
(3)


Here, *M* is the number of fluorescent species in the confocal volume; *r* and *z* are radius and half height of the confocal volume, respectively, which is often assumed to have 3D Gaussian illumination profile [50]; τ_Di_ is 2D lateral diffusion time of fluorescent species *i* across the confocal volume; *f_i_* is the fraction of fluorescent species *i*. We used a single species fitting for free RsAqpZ-mBanana, whereas for proteoliposomes autocorrelation curves were fit to the two species model.

We initially followed a molecular brightness method based on FCS [51] to calculate number of RsAqpZ tetramers per liposome, N_RsAqpZ_. In this method, free RsAqpZ-mBanana tetramer in HEPES buffer with 1% OG was used as a standard. Molecular brightness (photons emitted per molecule at standard excitation rate) of RsAqpZ-mBanana, ε_RsAqpZ-mBanana_, was measured by dividing total number of collected photons throughout the experiment by the duration of the measurement and dividing that number by the number of RsAqpZ-mBanana in the confocal volume, N_RsAqpZ-mBanana_, as obtained by fitting of the time-shifted autocorrelation curve to a 3D diffusion model (Equation 3). The molecular brightness of the RsAqpZ-mBanana proteoliposomes, ε_lip_, was determined likewise. The ratio of the molecular brightness of proteoliposomes to the molecular brightness of the free RsAqpZ-mBanana,(ε_lip_/ε_RsAqpZ-mBanana_), in principle yield an estimate of the number of RsAqpZ-mBanana tetramers per liposome, N_RsAqpZ_. However, this method did not result in consistent numbers for RsAqpZ tetramers in proteoliposomes at a fixed protein to lipid ratio, likely due to significantly altered fluorescence lifetime of mBanana in detergent micelles versus an aqueous environment surrounding the proteoliposomes (Figure S5). Therefore, this method was not pursued further.

The inability to effectively use molecular brightness led us to develop an alternative approach in which the tagged proteins are resolubilized from the proteoliposomes to directly determine the number of RsAqpZ tetramers per liposome. This alternative FCS-based technique for counting membrane proteins in liposomes was recently published by others in the study of a sodium transporter [42], and avoids the problem of changing molecular brightness.

**Figure 5:**
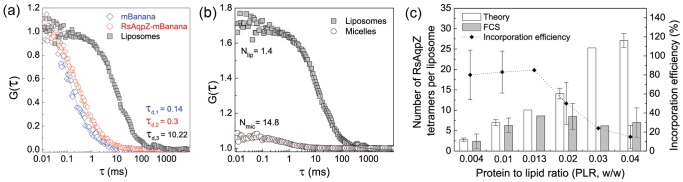
Determination of the stoichiometry and reconstitution efficiency of RsAqpZ in phospholipid vesicles. mBanana fusion of RsAqpZ was expressed and purified from *R. sphaeroides* and reconstituted in PC/PS vesicles at varying protein to lipid ratios. The number of RsAqpZ tetramers per liposome was evaluated by FCS, a) Normalized autocorrelation curves of mBanana species, mBanana fluorescent protein, RsAqpZ-mBanana protein tetramer and phospholipid vesicles embedded with RsAqpZ tetramer has significantly different diffusion times across the confocal volume, which can be inferred by the lag time required to reach the half of the value of the autocorrelation factor, τ_G(0)_/_2_. b) Autocorrelation curves for RsAqpZ-mBanana proteoliposomes before and after resolubilization in detergent 2.5% OG. A relatively high correlation coefficient for liposomes indicates a low number of particles in the confocal volume (N_lip_). For RsAqpZ-mBanana liposomes at PLR of 0.02, when detergent is added the diffusion time of the particles across the confocal volume decreased more than 18 times and the number of particles increased more than 10 times (N_prot_), implying effective resolubilization of RsAqpZ-mBanana. The autocorrelation curves were fit to a 3D diffusion equation (Equation 3) to obtain the concentration of the particles in the confocal volume. The stoichiometry of RsAqpZ tetramers per liposome (N_RsAqpZ_) was calculated taking the ratio of N_prot_ over N_lip_. c) Reconstitution efficiency of RsAqpZ-mBanana in phospholipid vesicles. The stoichiometry of RsAqpZ per liposome obtained from FCS was compared to the theoretically maximum value for each protein to lipid ratio (PLR) to evaluate the efficiency of reconstitution (details in File S1).

The time frames of the autocorrelation function, G(τ), represent the length of time that the fluorescent species spends within the confocal volume. Since the confocal volume has a precise dimension, this is then related to the diffusion time of the species, with larger species having longer diffusion times. As expected, the diffusion time of the tagged aquaporin in proteoliposomes is much longer than the free protein, and the increased size of the tagged protein (∼204 kDa in tetrameric form) relative to the mBanana fluorophore (27 kDa) is also readily observed (Figure 5). The diffusion time of the detergent-solubilized RsAqpZ-mBanana tetramer was slightly higher compared to the purified tetrameric protein fraction that was used to form the liposomes. This can be attributed to the observation that RsAqpZ-mBanana resolubilized from liposomes might have residual phospholipids and OG molecules surrounding the protein tetramers resulting in larger effective diffusing species. For the purpose of calculating single-channel permeability, the important observation is the 2 orders of magnitude reduction in diffusion time of the OG-solubilized versus liposome-incorporated RsAqpZ-mBanana. This demonstrates complete dissolution of the liposomes that were used for permeability measurements. We calculated the number of RsAqpZ-mBanana proteoliposomes in the confocal volume (N_lip_) by fitting the autocorrelation curves constructed from TCSPC data to a 2 species, 3D diffusion equation (Figure 5b) [51]. A triplet state model was used, when required.

Liposome samples were then solubilized with HEPES buffer with a final concentration of 2.5% OG releasing RsAqpZ-mBanana in detergent micelles, and the number of free aquaporin tetramers in the confocal volume (N_prot_) was likewise determined. Thus, the number of the RsAqpZ tetramers per liposome (N_RsAqpZ_) was calculated taking the ratio of N_prot_ over N_lip_, which then allows calculation of the incorporation efficiency based on the initial purified protein level used to produce the proteoliposomes (Figure 5c).

This resolubilization approach provided measurements of the aquaporin tetramer incorporation that correspond directly to proteoliposomes with known water permeability. We conducted FCS experiments with RsAqpZ-mBanana proteoliposomes at different protein to lipid ratios (PLR) and found the most sensitive experimental range to be between PLR 0.004 and PLR 0.04 for mBanana fusion of RsAqpZ. At lower PLR ratios resolution from FCS and stopped-flow experiments was lost due to low photon count and relatively high background permeability of PC-PS lipid vesicles. We evaluated the efficiency of protein incorporation into the phospholipid liposomes by comparing the number of RsAqpZ-mBanana tetramers per liposome obtained from FCS experiments and the theoretical number corrected for lipid displacement by assuming 100% RsAqpZ incorporation and the geometric characteristics of the liposomes, lipids and tetramers at a given PLR (described in File S1 and depicted in Figure S6). Stopped-flow light scattering experiments showed that the water permeability of proteoliposomes embedded with RsAqpZ-mBanana leveled out starting from PLR 0.02 (Figure 4), where incorporation efficiency was around 50% (Figure 5c).

### Water Channel Activity of RsAqpZ and EcAqpZ in ABA55 Polymer Vesicles

**Figure 6:**
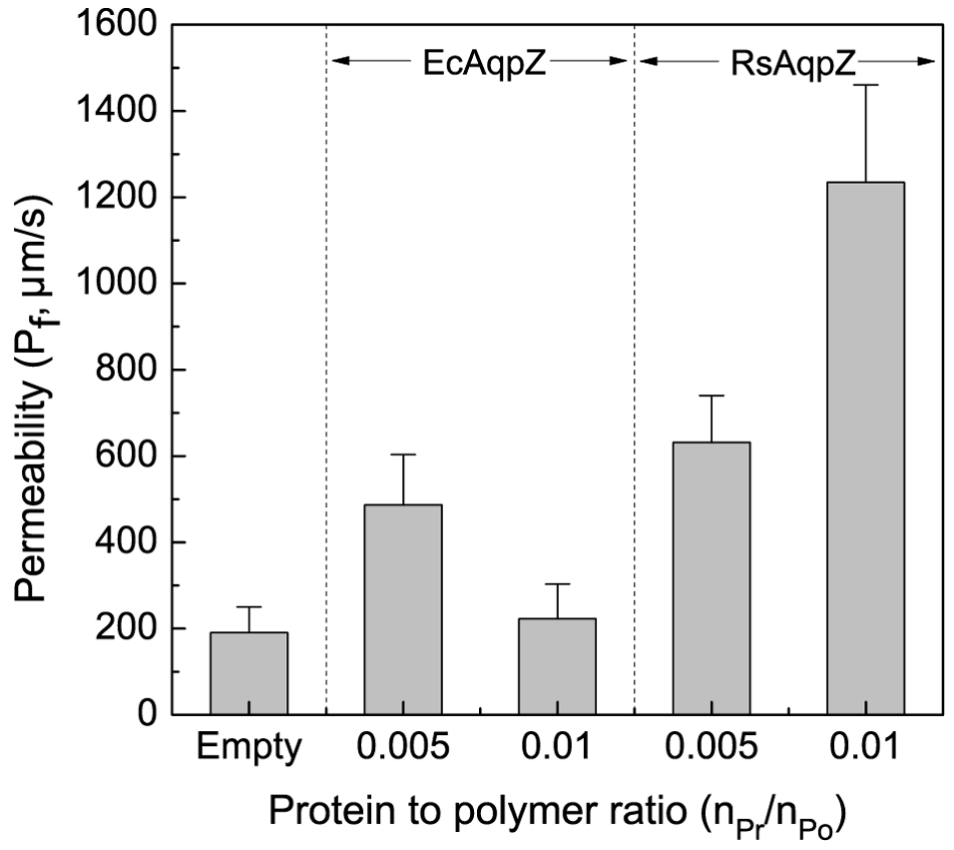
Water permeability of ABA55 vesicles embedded with EcAqpZ and RsAqpZ-mBanana. ABA55 polymersomes embedded with EcAqpZ and RsAqpZ-mBanana were prepared using film rehydration method and dialyzed for insertion of the proteins in polymer. Unilamellar polymersomes with a low polydispersity (PDI <0.25) were obtained using consecutive extrusions through 400 nm and 200 nm track-etched filters and were then used to measure water permeability by stopped-flow light scattering. EcAqpZ and RsAqpZ-mBanana remained active in ABA55 polymer vesicles. RsAqpZ-mBanana showed higher water permeability in part due to higher incorporation efficiency.

The water channel activity of EcAqpZ and RsAqpZ-mBanana was measured in ABA55 triblock copolymer vesicles and compared to polymersomes without these proteins. Both proteins showed water transport activity as evidenced by significantly higher permeability of proteopolymersomes as compared to control polymersomes (Figure 6). Water permeability of EcAqpZ proteopolymersomes actually reduced by 50% at a higher PrPR of 0.01 compared to PrPR 0.005 indicating a decrease in insertion efficiency. In contrast, RsAqpZ polymersomes showed a proportional increase in water permeability with PrPR, which is consistent with higher incorporation efficiency. The observation of no precipitation for RsAqpZ during dialysis qualitatively suggests improved incorporation of this protein into polymer membranes.

## Discussion

Aquaporins facilitate passive transport of water through membranes at extremely high rates (10^9^–10^10^ molecules per channel per second). Aquaporins from plant and animal species have well-established physiological roles. However, the function of microbial aquaporins is not well understood, as many species do not have identifiable aquaporins. Characterization of additional aquaporins will enable deciphering the function of this important class of proteins in microorganisms.

*Rhodobacter* species have a multiplicity of growth modes and have been extensively studied as model organisms in photosynthesis research. They have gained importance as novel expression platforms for membrane proteins due to their intracellular membranes providing large available membrane areas for recombinantly expressed membrane proteins [52]. Their use in bioremediation of heavy metal cations [52] and in biohydrogen production [53] is also proposed. For these reasons RsAqpZ could be a good target for characterization among other bacterial aquaporins. In this study, we cloned, and characterized RsAqpZ from *R. sphaeroides* ATCC 17023 that was homologously overexpressed under anaerobic photoheterotrophic growth conditions. We used a knockout strain that is incapable of synthesizing the second tier light-harvesting complex (LH-II) in the photosynthetic apparatus, which may comprise up to 10% of the actual biomass in this photosynthetic growth mode. Thus, more membrane area was available for recombinantly expressed membrane proteins. RsAqpZ was purified at 5 mg/L by metal affinity chromatography, although actual expression level was estimated to be around 20 mg/mL via western-Blot analysis. The purified protein was functional, which allowed assessment of channel mediated water transport rates by stopped-flow light scattering. We could determine the amount of protein incorporated in each liposome by using a fluorescently tagged version of RsAqpZ. From these measurements, we found the permeability of RsAqpZ to be ∼40×10^9^ H_2_O molecules per channel per second, which is the highest value reported thus far for aquaporins.

**Figure 7:**
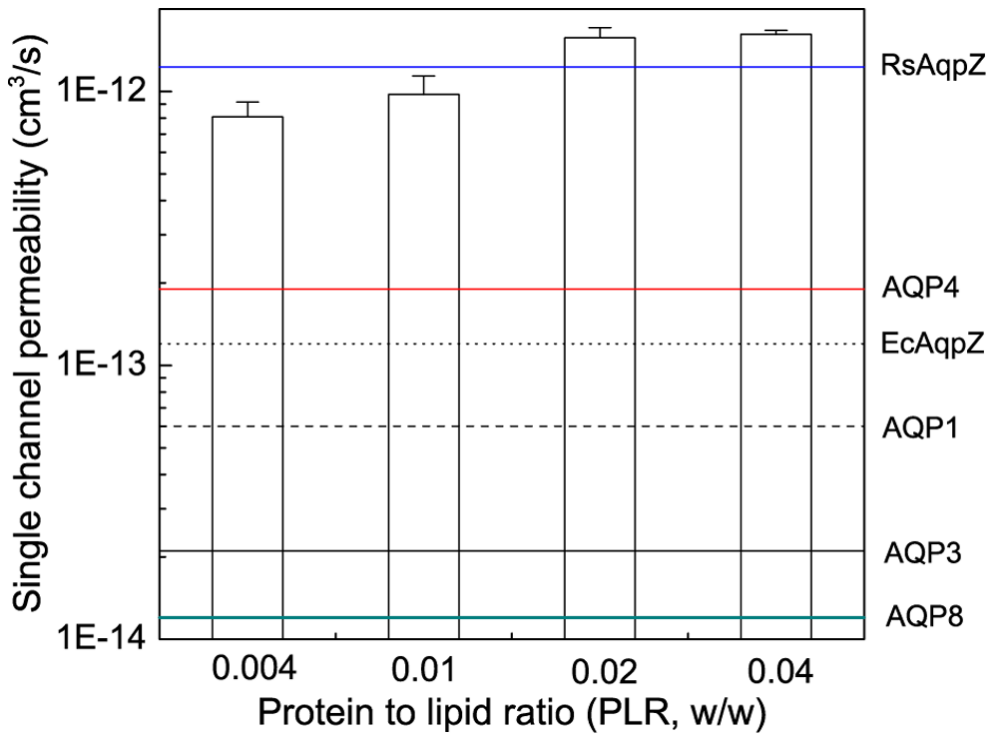
Single channel permeability of RsAqpz in proteoliposomes compared to single channel permeability of other reported aquaporins. The data on this figure includes permeability data for RsAqpZ (this study), AQP4 [54], EcAqpZ [20], AQP1 [55], AQP3 [48], AQP8 [56].

The single channel water permeability of RsAqpZ was calculated to be 1.23±0.43×10^−12^ cm^3^/s based on the average of 4 data points from PLR 0.004 to 0.04 (Figure 7). This value is roughly an order of magnitude higher single channel permeability than experimental (10×10^−14^ cm^3^/s) and MD simulation values (4.2×10^−14^ cm^3^/s) for EcAqpZ. However, previous *E.coli* aquaporin experimental permeability studies presumed 100% incorporation of the purified protein into the liposomes, which might lead to underestimation of the single channel permeability. Figure 7 compares the measured single- channel permeability of RsAqpZ with reported single channel permeability of human AQP1, human AQP3, human AQP4 and human AQP8. The results presented here show the importance of including incorporation efficiency into single-channel calculations, and indicate the utility of re-evaluating previously reported aquaporin permeability values to include this correction.

Incorporation of RsAqpZ into liposomes enhanced the water permeability by 18 times compared to empty liposomes and by 2.9 times compared to EcAqpZ proteoliposomes at a PLR of 0.02. Water permeability values measured in this study are higher than those reported by Borgnia et al for EcAqpZ. [20]. At lower PLR ratios, RsAqpZ and EcAqpZ proteoliposomes have comparable water permeability values. However, EcAqpZ proteoliposome water permeability plateaus around PLR = 0.02 (Figure 3). A similar behavior was observed for RsAqpZ, however at PLR = 0.04. These results imply that subunit permeability for RsAqpZ and EcAqpZ might be comparable and the difference may be due to an artifact of earlier studies not taking into account the insertion efficiency into liposomes. From this study, it seems that RsAqpZ incorporates into PC/PS lipid vesicles at a higher efficiency compared to EcAqpZ.

Energy of activation for water transport was determined as 2.93±0.6 kcal/mol for RsAqpZ proteoliposomes, is consistent with RsAqpZ mediated water transport rather than diffusive transport through lipid bilayers. This activation energy falls at the lower end of the reported range of activation energy values for other orthodox aquaporins. Water transport through RsAqpZ in proteoliposomes did not seem to be affected by pH below or above neutral pH (Figure S4).

Aquaporins have been proposed for use in biomimetic water desalination membranes to reduce energy costs in desalination processes [21]–[23], [39]. EcAqpZ is commonly used in these technological assessments due to its relatively high availability and selective water permeability. Here, we have shown that RsAqpZ has higher incorporation efficiency and potentially one order of magnitude higher subunit permeability compared to the published values for EcAqpZ in phospholipid vesicles. Moreover, water channel activity of RsAqpZ was retained in vesicles of ABA55 triblock copolymer where incorporation efficiency also seems to be higher compared to EcAqpZ. These findings suggest that RsAqpZ may be a viable alternative to EcAqpZ in biomimetic membrane technologies.

Understanding the physiological roles of bacterial aquaporins will require characterization of more aquaporins from different classes. In particular the physiological role of the RsAqpZ needs to be further explored through phenotype characterization of knockouts. To the best of our knowledge, this study represents the first study showing biophysical characterization of an orthodox aquaporin from a photosynthetic bacterium.

## Supporting Information

Figure S1 **Multiple cloning sites of the expression vectors for RsAqpZ and RsAqpZ-mBanana.**
(TIF)

Figure S2 **Increasing PLRs during EcAqpZ reconstitution into liposomes leads to increased precipitation.** a) Photograph of dialysis buttons prepared from a 3.8 mg/mL protein stock at increasing PLRs. b) Photograph of dialysis buttons prepared from a 0.66 mg/mL protein stock at increasing PLRs. c) Dialyzed samples at increasing PLRs after 18,000×g centrifugation show increasing amounts of precipitation. d) SDS page analysis of precipitates and supernatants from C show that precipitates are high molecular weight aggregates, which did not enter in the gel, whereas supernatant did not have a significant amount of aggregates.
(TIF)

Figure S3 **Electron microscopy of samples with increasing PLRs during EcAqpZ reconstitution into liposomes also shows increased precipitation and thus lowered incorporation of proteins.**
(TIF)

Figure S4 **Water permeability of RsAqpZ proteoliposomes (PLR = 0.01) at different pH levels.** RsAqpZ proteoliposomes were formed at pH = 7.4 by detergent dialysis and moved into the buffer solutions at pH between 5.0 and 8.0 to investigate the pH effect on water permeability. There was not a significant change in water permeability as evidenced by ANOVA.
(TIF)

Figure S5 **Fluorescence Lifetime of mBanana in different surroundings.** RsAqpZ-mBanana: in elution buffer, mBanana-TEV: RsAqpZ-mBanana treated with TEV and filtered through 30 kDa MWCO filter, PLR 0.004–0.04: RsAqpZ-mBanana in proteoliposomes.
(TIF)

Figure S6 **The calculation of the number of RsAqpZ tetramers per proteoliposome.**
(TIF)

Table S1 **Analysis of variance for water permeability at different pH values.**
(TIF)

File S1 **Supporting Information.**
(DOCX)
