# Supplementary figures and images for: Molecular Cloning, Overexpression and Characterization of a Novel Water Channel Protein from Rhodobacter sphaeroides
Source: PLoS One. 2014 Jan 31;9(1):e86830. doi: 10.1371/journal.pone.0086830 (PMC3909002; doi:10.1371/journal.pone.0086830)

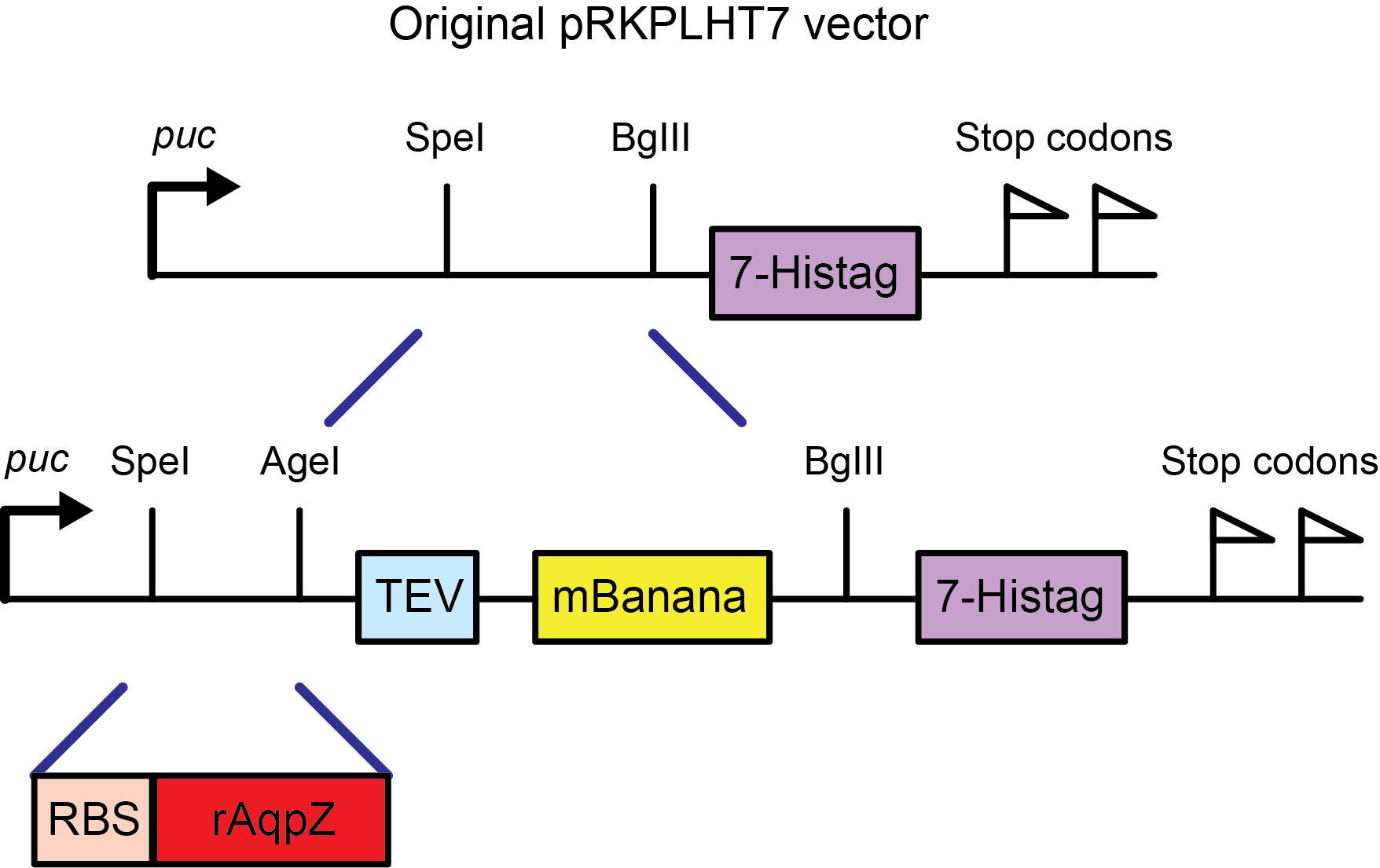

Supplement: Figure S1 — Multiple cloning sites of the expression vectors for RsAqpZ and RsAqpZ-mBanana. (TIF) [file pone.0086830.s001.tif]

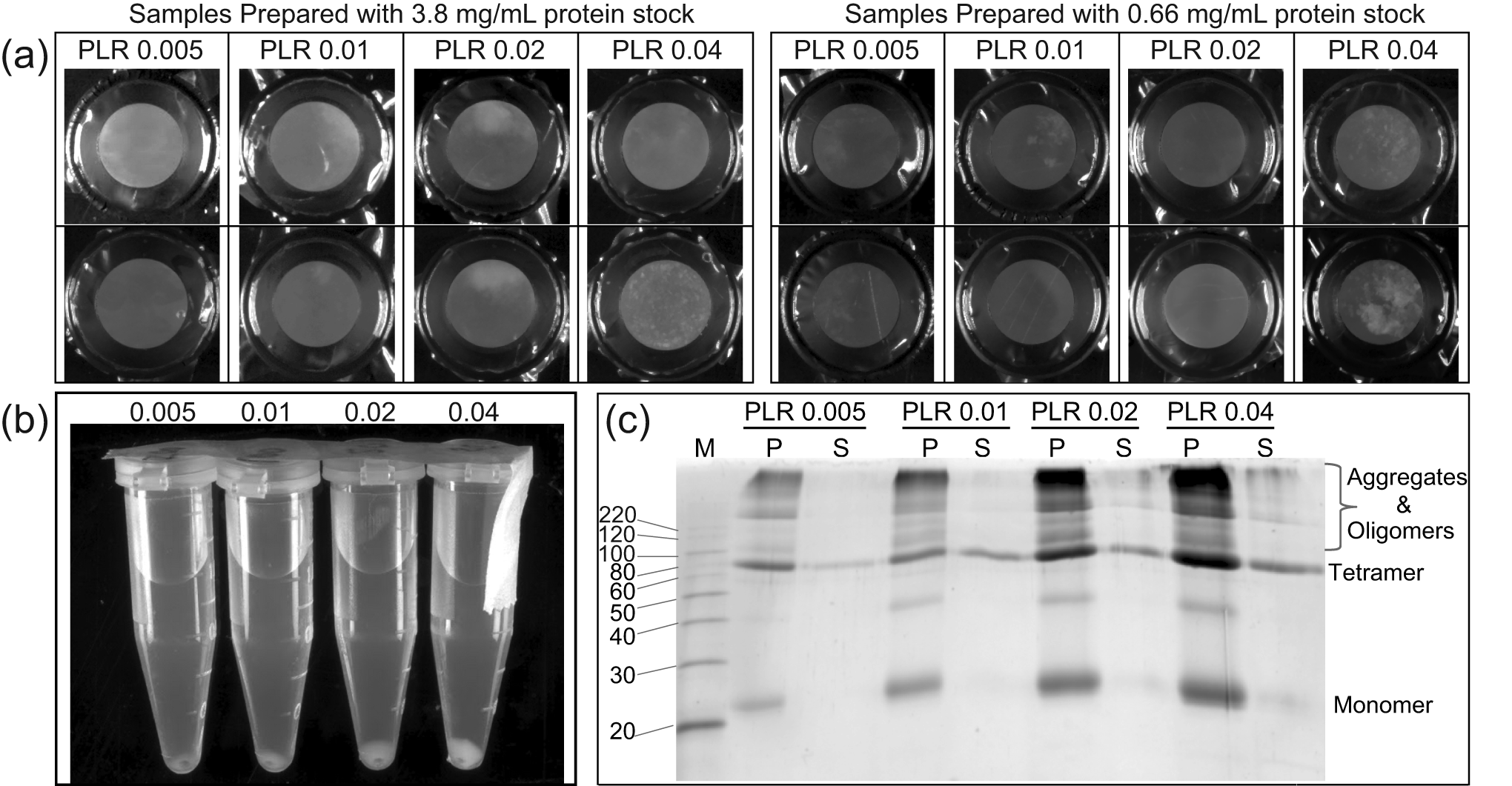

Supplement: Figure S2 — Increasing PLRs during EcAqpZ reconstitution into liposomes leads to increased precipitation. a) Photograph of dialysis buttons prepared from a 3.8 mg/mL protein stock at increasing PLRs. b) Photograph of dialysis buttons prepared from a 0.66 mg/mL protein stock at increasing PLRs. c) Dialyzed samples at increasing PLRs after 18,000×g centrifugation show increasing amounts of precipitation. d) SDS page analysis of precipitates and supernatants from C show that precipitates are high molecular weight aggregates, which did not enter in the gel, whereas supernatant did not have a significant amount of aggregates. (TIF) [file pone.0086830.s002.tif]

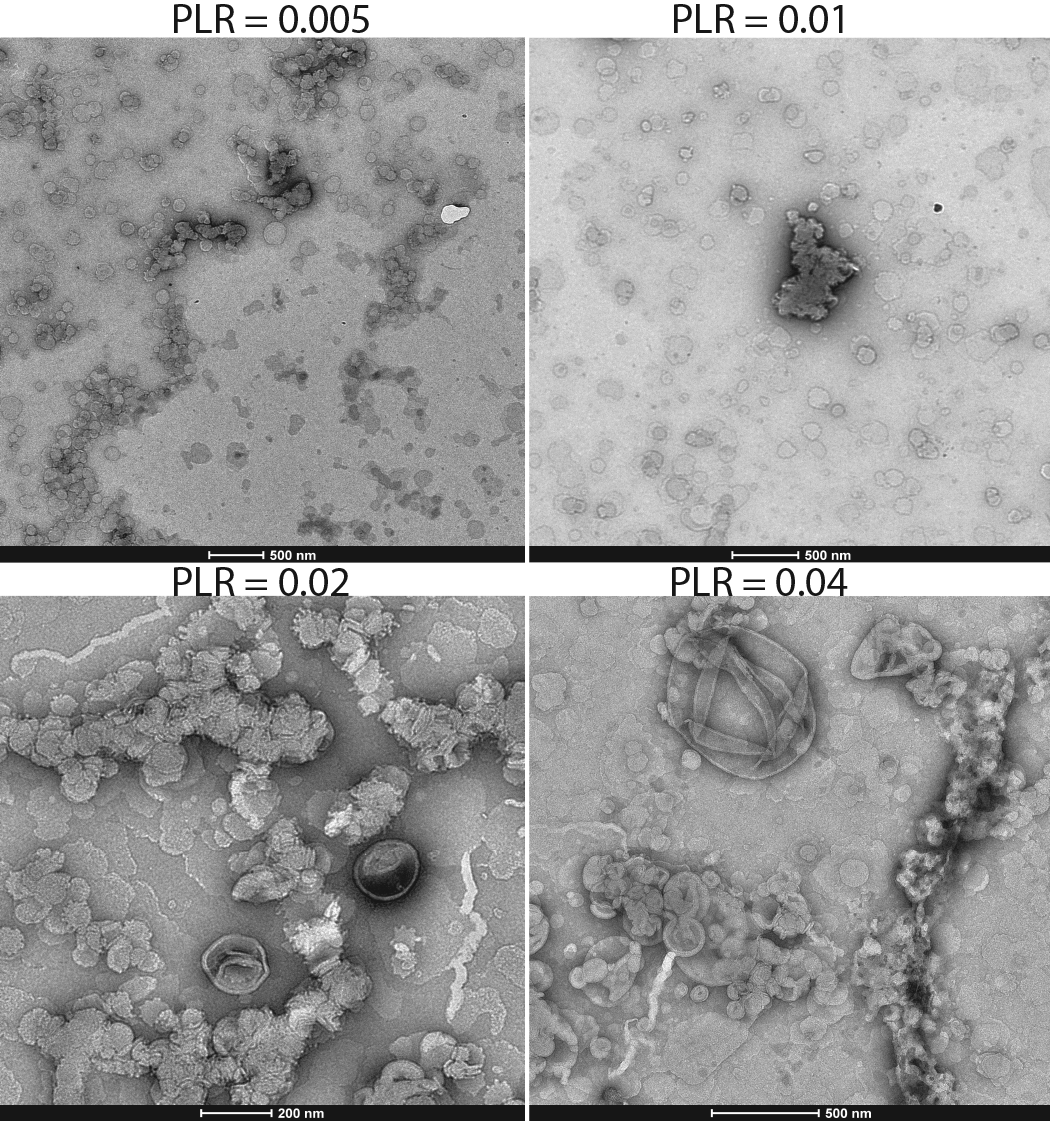

Supplement: Figure S3 — Electron microscopy of samples with increasing PLRs during EcAqpZ reconstitution into liposomes also shows increased precipitation and thus lowered incorporation of proteins. (TIF) [file pone.0086830.s003.tif]

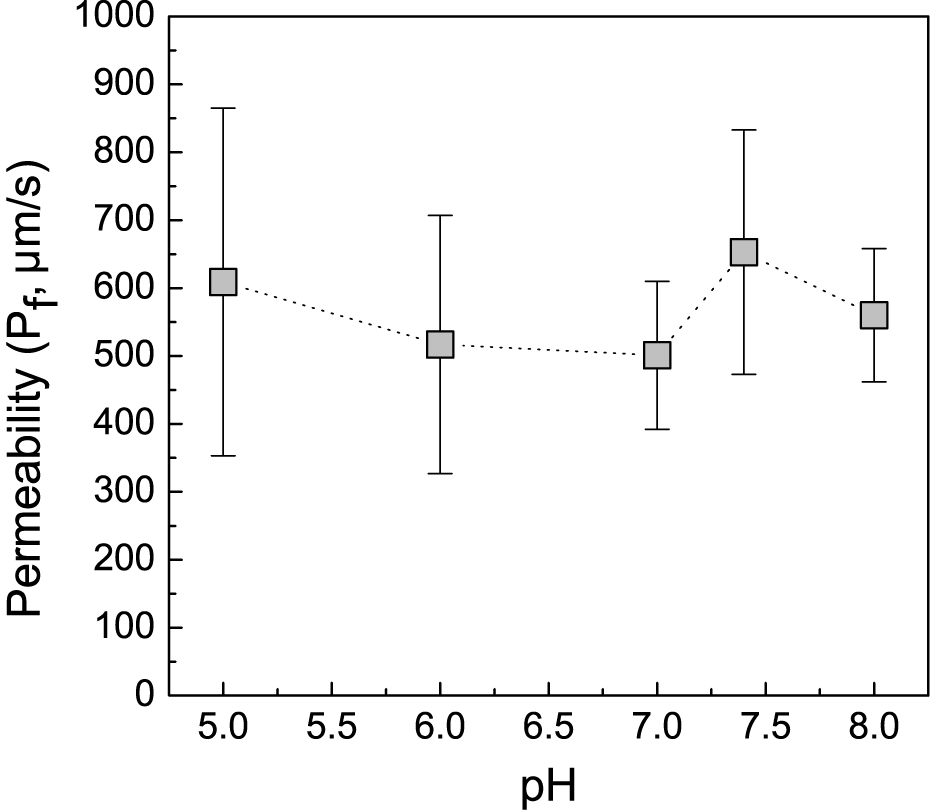

Supplement: Figure S4 — Water permeability of RsAqpZ proteoliposomes (PLR = 0.01) at different pH levels. RsAqpZ proteoliposomes were formed at pH = 7.4 by detergent dialysis and moved into the buffer solutions at pH between 5.0 and 8.0 to investigate the pH effect on water permeability. There was not a significant change in water permeability as evidenced by ANOVA. (TIF) [file pone.0086830.s004.tif]

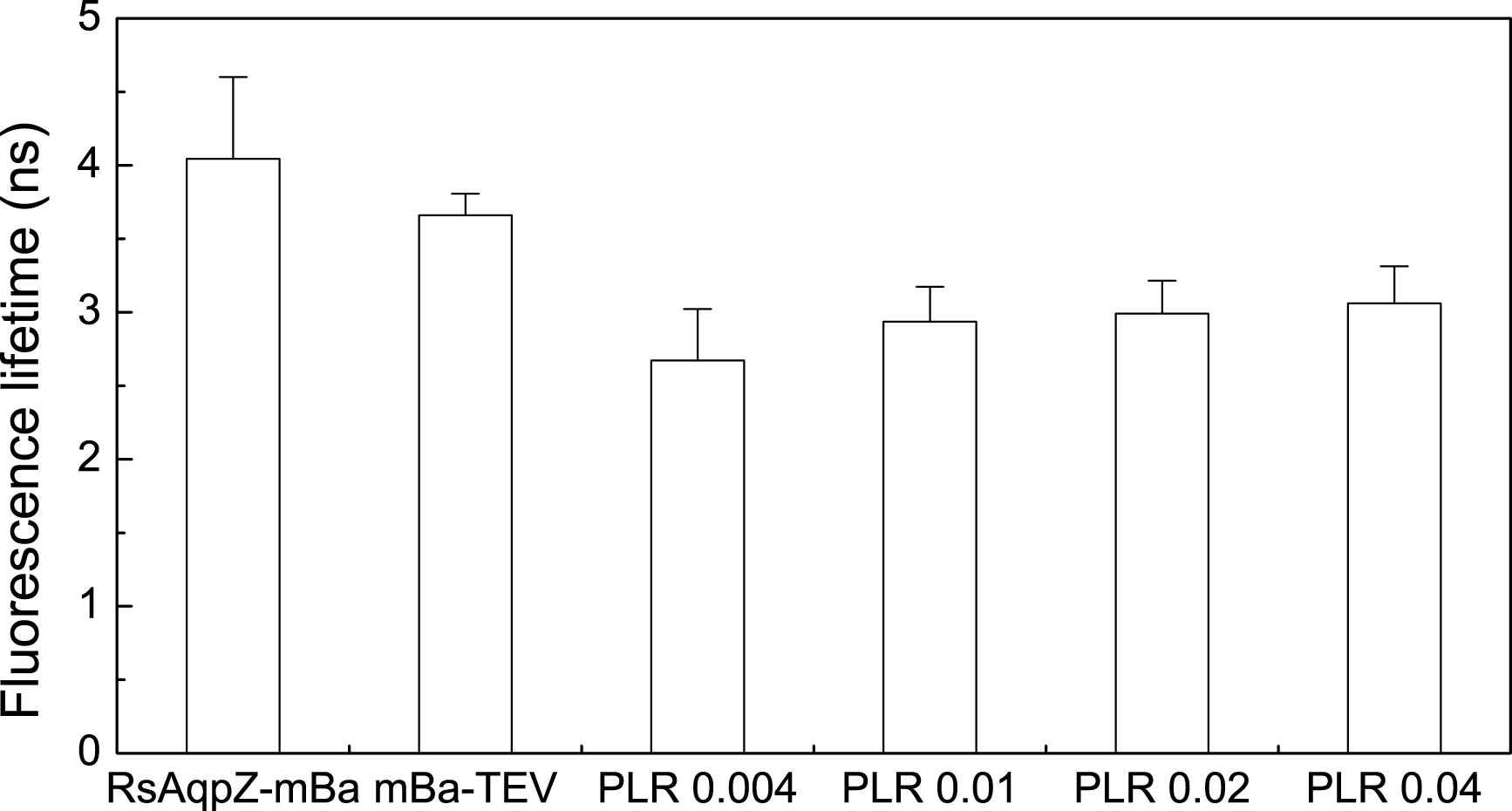

Supplement: Figure S5 — Fluorescence Lifetime of mBanana in different surroundings. RsAqpZ-mBanana: in elution buffer, mBanana-TEV: RsAqpZ-mBanana treated with TEV and filtered through 30 kDa MWCO filter, PLR 0.004–0.04: RsAqpZ-mBanana in proteoliposomes. (TIF) [file pone.0086830.s005.tif]

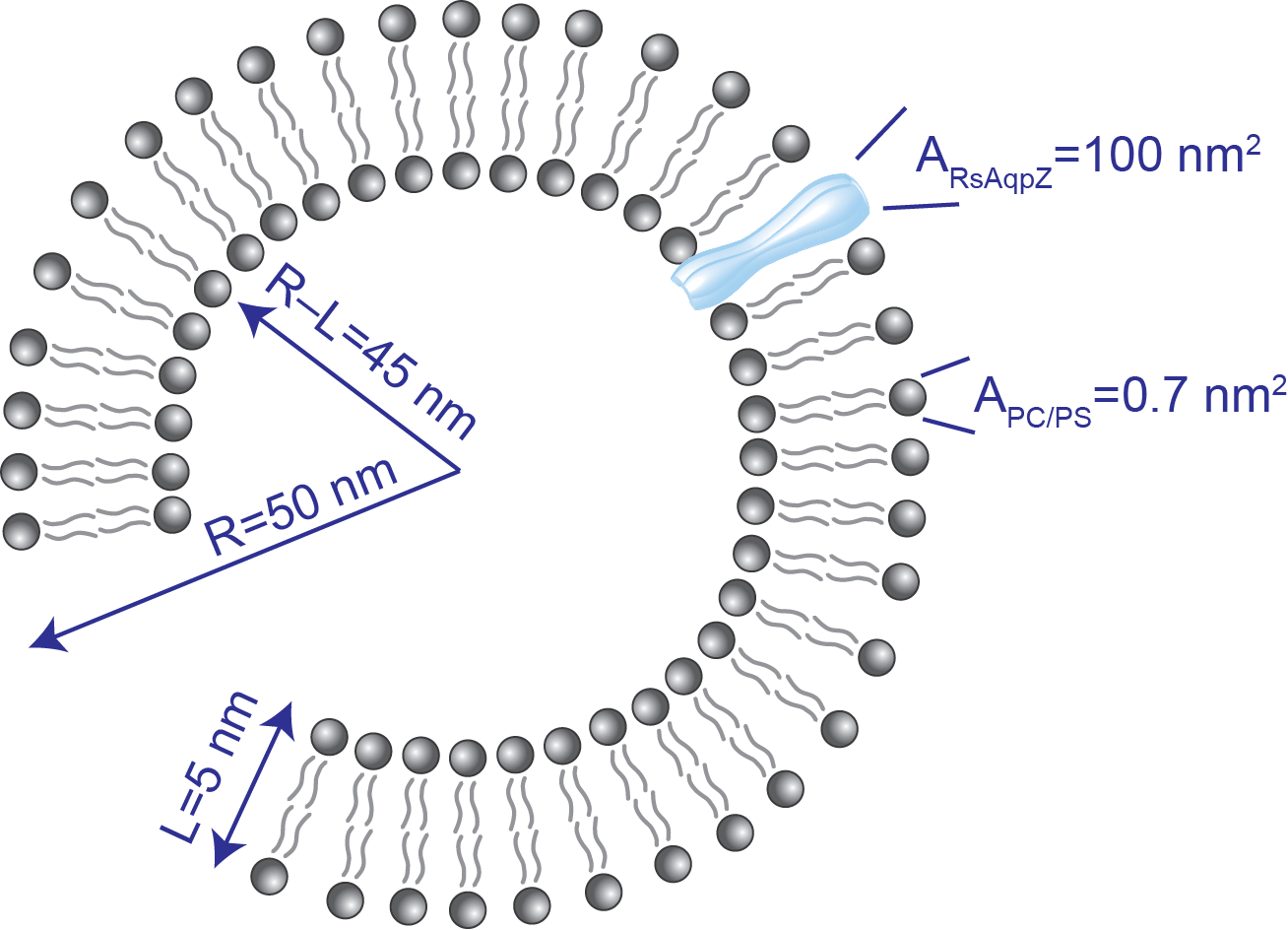

Supplement: Figure S6 — The calculation of the number of RsAqpZ tetramers per proteoliposome. (TIF) [file pone.0086830.s006.tif]
