# Supplementary material for: Molecular Cloning, Overexpression and Characterization of a Novel Water Channel Protein from Rhodobacter sphaeroides
Source: PLoS One. 2014 Jan 31;9(1):e86830. doi: 10.1371/journal.pone.0086830 (PMC3909002; doi:10.1371/journal.pone.0086830)
Supplement: File S1 — Supporting Information. (DOCX) [file pone.0086830.s008.docx]

## File S1: Supplementary Information

**Molecular Cloning, Overexpression and Characterization of a Novel Water Channel Protein from *Rhodobacter sphaeroides***

Mustafa Erbakan^1^, Yue-xiao Shen^2^, Mariusz Grzelakowski^3^, Peter J. Butler^1^, Manish Kumar^2,*^, Wayne R. Curtis^1,2,*^

^1^  Department of Biomedical Engineering, Pennsylvania State University, University Park, Pennsylvania, United States of America

^2^ Department of Chemical Engineering, Pennsylvania State University, University Park, Pennsylvania, United States of America

^3^ AquaZ Inc, Cincinnati, OH

* corresponding authors: manish.kumar@psu.edu and wrc2@psu.edu

### Text S1. Multiple Cloning Site of *pRKPLHT7-mBanana* Vector

The expression of membrane proteins in *Rhodobacter* has been achieved from a broad-host range vector, *pRKPLHT7* [26]. We modified this vector to create C-terminal mBanana fusion of RsAqpZ to use in FCS experiments. A linker sequence encoding for recognition site for highly specific tobacco etch virus (TEV) protease was placed between RsAqpZ and mBanana genes for site-specific cleavage of mBanana and 7-His from RsAqpZ.

### Text S2. Direct qualitative evaluation of decrease in insertion efficiency and protein precipitation at high PLRs for EcAqpZ

In order to provide a direct experimental verification to our claim that insertion efficiency is seldom 100% for membrane proteins, when incorporated in liposomes, for particularly at high PLRs, we conducted a series of experiments to show that visual precipitation is an indicator of imperfect insertion in such systems. We prepared two sets of samples with PC/PS lipids and EcAqpZ from two batches of protein stocks with concentrations of 3.8 mg/mL and 0.66 mg/mL. The total lipid concentration was kept constant at 6mg/mL for all samples, while protein concentration was adjusted so that samples had PLRs of 0.005, 0.01, 0.02 and 0.04. Figures S5A and B show photographs of the dialysis buttons used for conducting the experiments after completion of dialysis. They clearly show varying amounts of precipitation with an increase seen at higher PLRs. These samples were then collected in centrifuge tubes (corresponding samples shown in Figure S5A and B were combined) and subjected to centrifugation at 18,000xg. The precipitates should consist of non-membranous samples while the supernatant should be proteoliposomes. Figure S5C clearly shows that higher amounts of precipitates are obtained at higher PLRs indicating increasing unincorporated protein amounts in these samples. We ran both precipitate and supernatant samples on a 12% SDS-PAGE gel. Precipitates were resuspended in 100 μL dialysis buffer, after being washed with 1 ml dialysis buffer. Of this sample, a 16 μL aliquot was mixed with SDS sample buffer and loaded onto 12% acrylamide gel. 16 μL supernatant was mixed with SDS sample buffer and loaded on the same gel. Figure S5D shows the results from this analysis and confirms that precipitates included higher order oligomers of EcAqpZ as expected while supernatant samples only had the monomeric and tetrameric forms.

Electron microscopy was used to look at the samples in Figure S5A directly after harvesting and also indicates and increasing amount of precipitate (and thus lower incorporation) at increasing PLRs. These are shown in Figure S6.

### Text S3. pH Effect on Water Permeability of RsAqpZ

Water permeability of RsAqpZ proteoliposomes was tested at a pH range between 5 and 8.0. There was not a notable change in water permeability at different pH values consistent with other bacterial aquaporins [20]. A one-way analysis of variance (ANOVA) has been performed for water permeability data collected at different pH values using a web-based statistics program. p value was calculated as 0.81 depicting not a statistically significant difference between water permeability at different pH values.

### Text S4. Effect of Surroundings on Fluorescence Lifetime

Molecular brightness method was the initial attempt to determine the RsAqpZ stoichiometry per proteoliposome at altering PLR. In this method, molecular brightness of affinity purified RsAqpZ-mBanana was determined by counting the total number of photons via TCSPC module and fitting autocorrelation curve to a 3D diffusion model to obtain number of particles in the observation volume. Molecular brightness of the RsAqpZ-mBanana embedded proteoliposomes was calculated likewise. If the molecular brightness of RsAqpZ-mBanana in the stock solution and lipid bilayer remained the same, the number of RsAqpZ tetramers per proteoliposome could have been determined by taking ratio of the molecular of proteoliposome over free RsAqpZ-mBanana. Although a positive correlation between the measured water permeability levels from stopped-flow experiments and RsAqpZ-mBanana stoichiometry per proteoliposome from FCS experiments was obtained, it seemed that RsAqpZ-mBanana stoichiometry was significantly underestimated compared to the theoretical stoichiometry (described in Supplementary Text 5). This implied that there could be a significant change in molecular brightness value of RsAqpZ-mBanana in elution buffer versus lipid microenvironment. Indeed, we noted a dramatic cessation in fluorescence lifetime for RsAqpZ-mBanana in proteoliposomes compared to the free form (Figure S5), which indicated a significant change in molecular brightness and invalidated the utilization of free RsAqpZ-mBanana as standard.

### Text S5. Calculation of Single Channel Permeability

Initial total lipid concentration in the ternary mixture of PC/PS (4:1 molar ratio), RsAqpZ and HEPES buffer was 6 mg/ml prior to dialysis, extrusion and size exclusion steps. This number was kept constant for all samples, while RsAqpZ concentration was adjusted according to the desired protein to lipid ratio (PLR). Calculation of RsAqpZ tetramers per liposome involves determination of total surface area of PC/PS lipids and RsAqpZ tetramers in the initial ternary mixture and scaling it down to a hypothetical liposome (Figure S6). For example, a sample at PLR 0.01, has 6 mg/mL PC/PS and 0.06 mg/mL in the initial sample mixture. Considering mole-averaged molecular weight of PC/PS mixture is 781 g/mol, total number of PC/PS molecules in initial mixture is 4.62x10^18^. Likewise, considering RsAqpZ-7His tetramer has a molecular weight of 100 kDa, total number of RsAqpZ tetramers in the initial mixture is 3.61x10^14^. Averaged head group area of a phospholipid is 0.7 nm^2^, while an RsAqpZ tetramer has an estimated cross-sectional area of ~100 nm^2^ parallel to the lipid bilayer. Thus, total lipid head group area and total cross-sectional area of protein facing inside and outside the proteoliposomes can be calculated as 3.24x10^18^ and 7.22x10^16^, respectively. This yields an areal ratio of 44.8 (lipid/protein), which should be maintained in proteoliposomes, as well. Considering a 5 nm lipid bilayer thickness, sum of inner and outer surface area of a proteoliposome with 100 nm outer diameter is 56863 nm^2^. Surface area contributed to this number by protein is calculated as 1241 nm^2^, which corresponds to 6.2 RsAqpZ tetramers/liposome for a PLR of 0.01.
